# The Water Extract of *Juniperus communis* L. Induces Cell Death and Sensitizes Cancer Cells to Cytostatic Drugs through p53 and PI3K/Akt Pathways

**DOI:** 10.3390/ijms20092054

**Published:** 2019-04-26

**Authors:** Atso Raasmaja, Ulla Stenius, Aram Ghalali

**Affiliations:** 1 Division of Pharmacology & Pharmacotherapy, Faculty of Pharmacy, University of Helsinki, P.O. Box 56, 00014 Helsinki, Finland; 2 Institute of Environmental Medicine, Karolinska Institutet, P.O. Box 210, 17177 Stockholm, Sweden; ulla.stenius@ki.se (U.S.); aram.ghalali@ki.se (A.G.)

**Keywords:** cell death, plant extract, *Juniperus communis* L., p53, Akt, A549 non-small lung cancer cells

## Abstract

Juniper (*Juniperus communis* L.) is a northern coniferous plant generally used as a spice and for nutritional purposes in foods and drinks. It was previously reported that juniper extract (JE) affects p53 activity, cellular stress, and gene expression induced cell death in human neuroblastoma cells. Therefore, the effects of juniper on p53 and Akt signaling was examined further in A549 lung, 22RV1 and DU145 prostate, and HepG2 liver cancer cells using Western blot, confocal microscopy, and MTT analysis. We found that juniper simultaneously decreased cell viability, activated the p53 pathway, and inactivated the PI3K/Akt pathway. The p53 activation was associated with increased nuclear p53 level. Akt was dephosphorylated, and its inactivation was associated with increased levels of PHLPP1 and PHLPP2 phosphatases. Parallel increases of PARP suggest that JE decreased cell viability by activating cell death. In addition, JE potentiated the effects of gemcitabine and 5-fluorouracil anticancer drugs. Thus, JE can activate cell death in different cancer cell lines through p53 and Akt pathways.

## 1. Introduction

Plant-derived phytochemicals have been widely studied as compounds for potential nutritional and health-promoting strategies. Juniper (*Juniperus communis* L., Cupressaceae) is a northern coniferous plant, and its berries have been used as a spice and for other purposes. Since juniper extract (JE) contains many phenolic compounds, its potential physiological effects could be mediated through their specific bioactive properties. In previous studies it has been found that JE influences diverse cellular functions such as p53 activity, cellular stress, and gene expression, and furthermore induced cell death in human neuroblastoma cells [1,2,3]. 

The p53 and PI3K/Akt signaling pathways are two key regulators of cell survival and apoptosis, and their impaired functions are linked to many types of cancers [4]. The tumor suppressor protein, p53 is active in the cytosol and nucleusand controls many cellular proteins as well as gene expression [5,6]. p53 acts onprotein phosphorylation/dephosphorylation and gene acetylation/deacetylation through direct cytosolic effects and nuclear translocation. The activity of Akt is regulated by phosphatidylinositol-3-kinase (PI3K) as a part of the plasma membrane-linked second messenger system [7,8]. The production of phosphatidylinositol 3,4,5-trisphosphate (PIP3) by PI3K leads to Akt phosphorylation. Akt is active in its phosphorylated form and can then also translocate into the nucleus and control gene expression. Akt phosphorylation itself is regulated by PHLPP1 and PHLPP2 phosphatases, and their impaired function is linked to cancer [9,10].

Our aim was to study the molecular effects of Juniper berry extract on the regulation of cell death in A549 lung cancer, 22RV1 and DU145 prostate cancer, and HepG2 liver cancer cells. The effects of JE were specifically analyzed on the levels of cell viability, p53, pAkt, PHLPP1/2, pGsk3Ser9 and PARP cleavage. In addition, the effects of JE were studied in combinations with two anticancer drugs, gemcitabine and 5-fluorouracil.

## 2. Results

### 2.1. Juniper Extract Decreased Cell Viability in Several Cancer Cell Lines 

**Figure 1:**
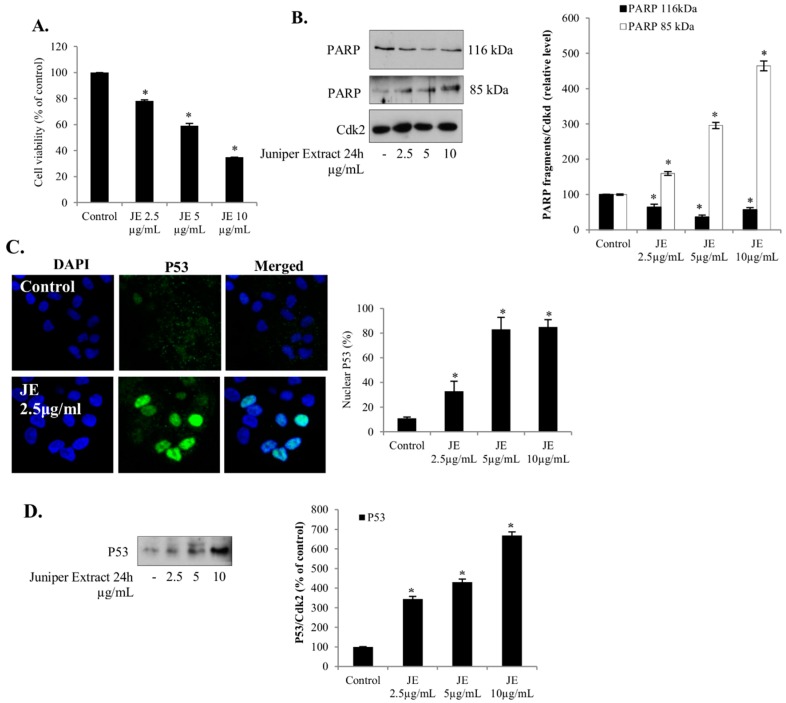
Juniper extract (JE) decreased cell viability, and induced the activation of P53 and apoptosis in non-small lung cancer A549 cells. Cells were grown as described in the Materials and Methods section, then starved for 24 h and incubated with JE for 24 hours. (**A**) Cell proliferation was estimated by MTT-assay. (**B**) Cell lysates were analyzed for non-cleaved PARP (116 kDa) and cleaved PARP (85 kDa) by Western blotting. Cdk2 was used as a loading control. (**C**) Cells were fixed and stained for P53 and analyzed by confocal microscopy (magnification: 63×). Signals were quantified and presented as a percentage of control. (**D**) Cell lysates were analyzed for P53 by Western blotting. Fig. **B** and **D** re from the same experiments, and the Cdk2 loading control is shown in (**B**). Data are presented as bars ± standard deviation from three independent experiments. * Significantly different from control, *p* ≤ 0.05.

The effects of JE on cell viability were analyzed in A549 non-small lung cancer, 22RV1 and DU145 prostate cancer, and HepG2 hepatocellular carcinoma cell lines. We found that JE caused a dose-dependent decrease in the cell viability with similar sensitivity across all these cancer cell lines (Figure 1A and Figure 3A–C). In order to understand the causes of decreased cell viability, we investigated whether these effects of JE were mediated by the activation of and mechanisms involved in cell death (e.g., p53 and pAkt pathways). 

### 2.2. Juniper Extract Activated the p53 Pathway in Parallel with the Inhibition of Cell Proliferation 

Changes in p53 protein and PARP cleavage are often used to detect apoptosis, and the nuclear localization of p53 is thought to be crucial for its proapoptotic properties [11]. Here, a dose-dependent increase in the level of cleaved PARP and a decrease in uncleaved PARP was detected when the A549 cells were treated with JE (Figure 1B). Next, we also studied the effects of JE on p53 levels and nuclear translocation in the non-small lung cancer A549 cells. We found that JE increased the level of p53 (Figure 1D) and induced the nuclear translocation of p53 (Figure 1C). The effects on the p53 and PARP occurred in parallel with the decreased cell number, indicating an active role in cell death.

### 2.3. Juniper Extract Inactivated the Akt Pathway 

**Figure 2:**
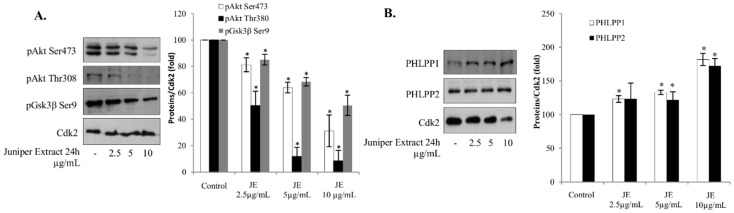
JE inhibited Akt signaling pathway in A549 cells. Serum-starved A549 cells were treated with JE for 24 h. (**A**) Cell lysates were analyzed for pAkt Ser473, pGSK3β Ser9, and pAkt Thr308 by Western blotting. (**B**) Cell lysates were analyzed for PHLPP1 and PHLPP2 by Western blotting. (**A**,**B**) Cdk2 was used as loading control. Data are presented as bars ± standard deviation from three independent experiments. * Significantly different from control, *p* ≤ 0.05.

It is known that Akt protects cells against apoptosis and hampers cancer therapy. The effect of JE on the level of pAkt, an activated form of Akt, was studied in A549 cells. We found that JE decreased the phosphorylation of Akt both at Serine 473 (Ser473) and Threonine 308 (Thr308) residues in a dose-dependent manner (Figure 2A). The phosphorylation of the Akt downstream target pGsk3β was inhibited, confirming that the JE decreased Akt-signaling (Figure 2A). Furthermore, JE seems to regulate Akt through targeting its phosphatases, PHLPP1 and PHLPP2. JE increased the levels of both phosphatases in a manner that correlated with the decreases of pAkt levels (Figure 2B). All these results are in line with the decreased activity of Akt pathway and correspond to an increased level of cell death pointing out an important role of Akt in the JE-induced cell death. 

### 2.4. Effects of Juniper Extract on Prostate and Hepatocellular Carcinoma Cells

**Figure 3:**
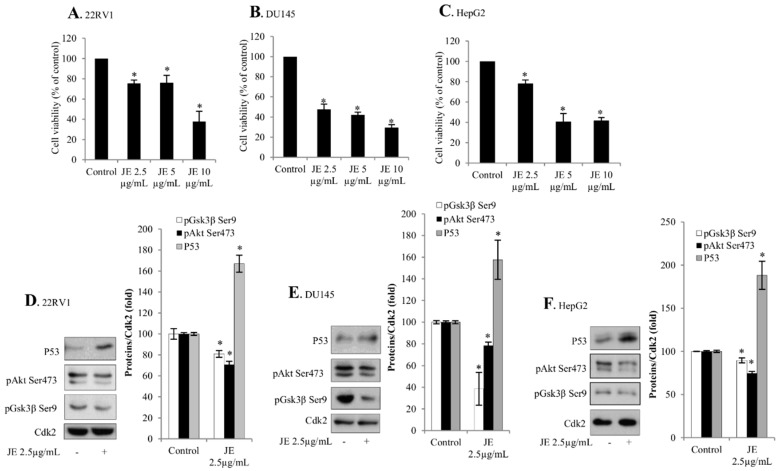
JE decreased both pAkt levels and cell proliferation in prostate and liver cancer cells. Serum-starved 22RV1, DU145, and HepG2 cells were treated with JE for 24 h. (**A**–**C**) Cell proliferation was estimated by MTT assay. (**D**–**F**) Cell lysates were analyzed for pAkt Ser473, pGsk3β Ser9, and P53 by Western blotting. Cdk2 was used as loading control. Data are presented as bars ± standard deviation from three independent experiments. * Significantly different from control, *p* ≤ 0.05.

The effects of JE were studied further in prostate and hepatocellular cancer cells in order to investigate the generality of the juniper-induced mechanisms and activation of cell death. It was shown that the juniper treatment also increased the level of p53 in 22RV1 and DU145 prostate cancer and HepG2 hepatocellular carcinoma cells (Figure 3D–F). In line with the anti-proliferative effects (Figure 3A–C), JE decreased both pAkt and pGsk3β levels in 22RV1 and DU145 prostate cancer cells and HepG2 hepatocellular carcinoma cells (Figure 3D–F). 

### 2.5. Juniper Extract Sensitized A549 Small Lung Cancer Cells to 5-Fluorouracil and Gemcitabine

**Figure 4:**
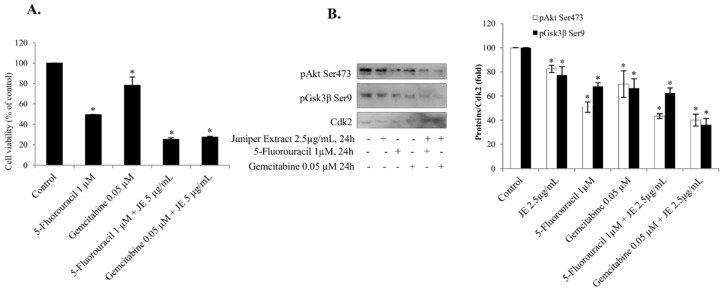
JE decreased pAkt and increased the effect of gemcitabine and 5-fluorouracil in A549 cells. Serum-starved A549 cells were treated with JE and/or gemcitabine or 5-fluorouracil for 24 h. (**A**) Cell proliferation was estimated by MTT assay. (**B**) Cell lysates were analyzed for pAkt Ser473 and pGSK3β Ser9 by Western blotting. Cdk2 was used as loading control. Data are presented as bars ± standard deviation from three independent experiments. *Significantly different from control, *p* ≤ 0.05.

The interaction of JE-mediated mechanisms with other cell death pathways was studied using 5-fluorouracil and gemcitabine, which have been used as anticancer drugs to treat in lung and pancreatic cancers. To determine whether JE enhanced the efficiency of these compounds, we investigated the effects of JE on cell viability and pAkt activation induced by 5-fluorouracil and gemcitabine in the A549 cells. The decreases in the cell viability by 5-fluorouracil and gemcitabine alone were associated with the decreases of pAkt and its downstream target pGsk3β (Figure 4A,B). When the cells were treated with a combination of JE and anticancer drugs, the decreases of both pAkt and pGsk3β were significantly potentiated, suggesting an interaction between 5-fluorouracil/gemcitabine and juniper on the pAkt pathway.

## 3. Discussion

Our data show that in low concentrations JE inhibited the phosphorylation of Akt in several cancer cell lines. We also present results showing that juniper sensitized cells to cytostatic drugs. Recent reports have suggested that the combined use of different drugs and natural products would improve the efficiency of cancer therapy. For example, a potentiation of apoptosis was found in tumorigenic cells with the combination of statins and etoposide, doxorubicin, or 5-fluorouracil [12,13]. Furthermore, statin treatment increased the sensitivity to etoposide and 5-fluorouracil in A549 non-small lung cancer cells [14]. It was suggested that the statin-induced sensitivity of apoptosis was mediated by the inhibition of Akt phosphorylation and nuclear translocation. Similarly, increased apoptosis has been found with the use of natural compounds and extracts. For example, the antitumor effects of wine and rosemary treatments in cancer cells could be mediated by the combined action of various compounds [15,16]. Earlier studies have shown in particular that the juniper berry extract activated cell death by increasing p53 amount and nuclear translocation in the human SH-SY5Y neuroblastoma cells [3]. In the present study, the anticancer effects of JE were seen in parallel with p53 activation and pAkt inactivation, suggesting that the extract promotes cell death by regulating both of these pathways, and possibly by other means as well. 

A composition analysis of the JE identified several phenolic compounds which were further classified to five main groups comprising flavones, flavonols, phenolic acids, flavanols, and biflavonoids [1,2,3,17]. The most abundant phenolic compounds were rutin, apigenin, isoscutellarein, hypolaetin, protocatechuic acid, and rutin. Here, the effective concentrations, dose dependence, exposure times, and mechanisms were similar in all studied cancer cell lines, and were comparable with our earlier findings in neuroblastoma cells. Accordingly, it was postulated that the JE, consisting of a unique combination of phenolic compounds, targets cancer cells by specific apoptotic mechanisms. 

The present results proved that the ability of JE to activate cell death is a more general effect, as seen here in several types of cancer cells. In previous studies, JE was examined only in the human SH-SY5Y neuroblastoma cells showing increased p53 activity and cell death [1,3]. In the present study, the anticancer effects of JE were also found in A549 non-small lung cancer A549, 22RV1 and DU145 prostate cancer, and HepG2 hepatocellular carcinoma cells. Interestingly, the effects of JE were observed at remarkably small amounts with even 100–1000× lower concentrations than those used for the respective compounds alone. The identified phenolic compounds were present at nanomolar concentrations in JE treatments [3]. In general, when used alone, pure phenolic compounds have been effective in decreasing cell viability or activating cell death at micromolar concentrations. This has been shown, for example, for apigenin and curcumin in SH-SY5Y neuroblastoma cells [1,18,19] or naringenin and quercetin in A549 small lung cancer cells [20,21]. Therefore, we propose that the effects of JE were due to the potentiation, synergy, or both between several plant polyphenols through common or different mechanisms. 

The PI3K/Akt signaling pathway is frequently up-regulated in human cancers, and is induced by cytostatic drugs. Our results show that JE inhibited the PI3K/Akt pathway by inhibiting its phosphorylation. Threonine 308 and Serine 473 were dephosphorylated, resulting in an inactivation of the Akt pathway, since the full activation of the Akt pathway is dependent on Akt phosphorylation at Threonine 308 and the phosphorylation at Serine 473 helps to maintain the threonine-induced activation. This inhibition could be mediated by JE-induced increases in the levels of PHLPP1 and PHLPP2 phosphatases. These phosphatases are responsible for Akt dephosphorylation [10]. Both PHLPP1 and PHLPP2 are tumor suppressors and downregulated in many cancers, thereby maintaining Akt activity as well as promoting proliferation, growth, and survival. In addition, the JE decreased the phosphorylation and activation of pGSK3β—a downstream target of Akt. 

Another important regulator of cell death is the p53 tumor suppressor. The p53 is mutated or inactivated in the majority of tumors. In the present study, the nuclear translocation of p53 and the ratio of cleaved/intact PARP were induced in parallel, demonstrating the activation of cell death in the A549 cells. The activation of p53 by the JE was also seen simultaneously with the inhibition of the Akt pathway in lung, hepatic, and prostate cancer cells. These and earlier results indicate that the activation of cell death is mediated through combined effects by juniper polyphenols [1,3]. Similar potentiation and interaction were also reported for statins sensitizing cancer cells to cytostatic drugs such as doxorubicin, 5-fluorouracil, and gemcitabine [12,13,14], and plant extracts or compounds [1,15]. Interestingly JE was also able to potentiate the antitumor effects of 5-fluorouracil and gemcitabine in A549 cells. The use of drug combinations may have synergistic therapeutic effects, improve selectivity, and decrease side effects [22]. 

It is suggested that the p53 activation and Akt inhibition are cell-death-promoting mechanisms of JE, and especially [23,24] together with cytostatic drugs, represent a potential model to study the mechanisms of drug combinations in cancer cells. The exact mechanisms of the present results are still unknown. Interactions of PI3K/Akt, p53, and PTEN activities in the regulation of apoptosis and drug sensitivity have been found, for example, in non-small-cell lung cancer cell lines [23,24]. Plant extracts and compounds have gained wide interest as a potential source for drug development against cancer and other diseases [25]. Thus, JE seems to have several amplifying targets to activate p53, inactivate the PI3K/Akt pathway, and reactivate cell death.

## 4. Materials and Methods

### 4.1. Plant Material and Reagents

Ripe and dried berries of juniper (*Juniperus communis* L.) cultivated for spice were commercially obtained from Paulig Ltd, Finland (http://www.pauliggroup.com). The extracts of ground juniper berries were prepared as described previously [17]. In brief, the extraction was performed by boiling water during a three-hour hydrodistillation, resulting in the removal of volatile oils. The aqueous extract was filtrated, freeze-dried, and stored at 4 °C. The yield of the extract was 422 mg/g of ground berries. The anticancer drugs 5-fluorouracil (5-Fu) and gemcitabine were purchased from Sigma-Aldrich (St. Louis, MO, USA) and Eli Lilly (Indianapolis, IN, USA), respectively. 

### 4.2. Cell Culture

The human carcinoma cell lines A549, DU145, 22RV1, and HepG2 were purchased from ATCC (Manassas, VA, USA). The A549 cell line is characterized by wild-type p53 and Akt genotype. DU145 has a mutated p53 and wild-type PTEN; 22RV1 has wild-type p53 and PTEN; and HepG2 has wild-type p53 gene and PTEN. A549 and DU145 cells were grown in DMEM, with 10% inactivated fetal bovine serum (FBS), and 1 mM sodium pyruvate. 22RV1 cells were grown in RPMI-1640 from Sigma-Aldrich (St. Louis, MO, USA) supplemented with 10% FBS. HepG2 cells were grown in MEM, with Earl’s salts and l-glutamine supplemented with 1 mmol/L of sodium pyruvate, nonessential amino acids, and 10% inactivated FBS. Media were supplemented with penicillin-streptomycin. Serum-starved cells were cultured with medium supplemented with 0.1% serum for 24 h. DMSO was used to dissolve 5-fluorouracil and gemcitabine at the final concentration of <0.4% when added to the cells. No effect of DMSO was observed.

### 4.3. Western Blotting

Cells were washed with PBS and lysed in IPB-7 buffer (triethanolamine-HCl (TEA), 1 M, pH 7.8; NaCl, 5 M; sodium deoxycholate, 4%; Igepal CA-630 or Nonidet P-40, 10%) with inhibitors (1 mg/mL phenylmethylsulfonyl fluoride, 0.1 mg/mL trypsin inhibitor, 1 mg/mL aprotinin, 1 mg/mL leupeptin, 1 mg/mL pepstatin, 1 mmol/L Na_3_–VO_4_, and 1 mmol/L NaF. Samples were subjected to SDS–PAGE and blotted onto a PVDF membrane (Bio-Rad, Hercules, CA, USA). The protein bands were probed using antibodies against Akt phosphorylated at residue Ser473 or Thr308, p53, PARP, and Cdk2 from Santa Cruz Biotechnology, Inc. (Santa Cruz, CA, USA); pGSK3β Ser9 from Cell Signaling (Beverly, MA, USA); PHLPP1 and PHLPP2 antibodies were from Bethyl Laboratories Inc. (Montgomery, TX, USA). Proteins were visualized with ECL procedure (Amersham Biosciences, Sweden). The results were analyzed with NIH Image 1.62 software. 

### 4.4. Confocal Microscopy

Cells were fixed with 4% formaldehyde, permeabilized with 0.2% Triton X-100 in 2% BSA buffer. Immunostaining was performed using mouse monoclonal p53 antibody. Secondary antibodies were conjugated with Alexa 488 (from ThermoFisher Scientific at NY, USA (Cat. Number: A11001). Cells were analyzed with a Zeiss LSM 510 META confocal laser scanning microscope (Zeiss, Oberkochen, Germany) equipped with a ×63 plan A oil-immersion lens. An argon laser was excited at 488 nm and fluorescence images were recorded from 500 to 550 nm. The localization of indicated proteins was measured by Zeiss LSM imaging software in multi-track mode.

### 4.5. MTT Assay

Cell viability was determined by 3-(4,5-dimethylthiazol-2yl)-2,5-diphenyltetrazolium bromide (MTT) assay detecting the cellular mitochondrial capacity to convert MTT tetrazolium salt to formazan. Cells were incubated with medium containing MTT (Sigma-Aldrich, St. Louis, MO, USA) for 4 h. The cells were then lysed in DMSO. The absorbance was measured at 570–620 nm.

### 4.6. Statistical Analysis

Statistical analysis was conducted using the Student’s *t*-test. The data were presented as mean ± SD. Experiments were performed at least three times with different batches of cells. Results were considered to be statistically significant at *p* ≤ 0.05. 

## 5. Conclusions

In conclusion, the JE containing several plant phenolic compounds activated cell death in a dose-dependent manner in A549 non-small lung cancer, HepG2 hepatocarcinoma, and 22RV1 and DU145 prostate cancer cells through inhibition of PI3K/Akt and activation of p53 pathways. This regulation was mediated by juniper’s effects on the Akt phosphorylation/dephosphorylation, PHLPP1 and PHLPP2, pGsk3 and intact/cleaved PARP, and p53 activation. The present results also showed that the JE has cell death promoting effects in several types of cancer cells from different origins and through specific target mechanisms. 

## Abbreviations

A549	Non-small lung cancer cell line
DMEM	Dulbecco’s modified Eagle medium
DMSO	Dimethyl sulfoxide
DU145	Prostate cancer cell line
HepG2	Hepatocellular carcinoma cell line
MTT	3-(4,5-Dimethylthiazol-2yl)-2,5-diphenyltetrazolium bromide
P53	Transcription factor
pAkt	Phosphorylated Akt
PARP	Poly (ADP-ribose) polymerase
PBS	Phosphate-buffered saline
pGsk3Ser9	Phosphorylated glycogen synthase kinase-3
PHLPP1/2	Phosphatases
PI3K	Phosphatidylinositol-3-kinase
PIP3	Phosphatidylinositol 3,4,5-trisphosphate
TEA	Triethanolamine-HCl
